# A giant popliteal lipoblastoma in a 23-month-old girl: a case report

**DOI:** 10.1186/s13256-017-1513-y

**Published:** 2017-12-05

**Authors:** Shogo Hashimoto, Kazutaka Kikuta, Tetsuya Sekita, Robert Nakayama, Shinichiro Takayama, Aya Sasaki, Kaori Kameyama, Masaya Nakamura, Morio Matsumoto, Hideo Morioka

**Affiliations:** 1 Department of Orthopaedic Surgery, Keio University School of Medicine, 35 Shinanomachi, Shinjyuku-ku, 160-8582 Tokyo, Japan; 2 Department of Orthopaedic Surgery, National Center for Child Health and Development, 2-10-1 Okura, Setagaya-ku, 157-8535 Tokyo, Japan; 3 Department of Pathology, Keio University School of Medicine, 35 Shinanomachi, Shinjyuku-ku, 160-8582 Tokyo, Japan

**Keywords:** Lipoblastoma, Children, Magnetic resonance imaging, Resection, Operative adaptation

## Abstract

**Background:**

Lipoblastomas are rare benign tumors that arise from embryonic white fat and almost always occur in babies and children. Here, we report a case of a giant popliteal lipoblastoma in a 23-month-old Japanese girl that was successfully treated via complete resection.

**Case presentation:**

Our patient was a 23-month-old Japanese girl. At 6 months of age, she presented at a nearby hospital with a mass on the popliteal side of her lower right leg. She had no symptoms and was diagnosed as having a benign adipose tumor via magnetic resonance imaging. The mass gradually increased in size, and she was referred to our hospital at 1 year and 11 months of age. A physical examination and radiology revealed a localized mass 13 × 10 × 7 cm in size in the aforementioned area that restricted knee movement and caused proximal tibia deformity. Magnetic resonance imaging showed a giant circumscribed subcutaneous mass with multiple partitions that was hyperintense on T1-weighted and T2-weighted images but not fat-saturated on T2-weighted images. Based on these findings, she was diagnosed as having a lipoblastoma. Because the mass surrounded her popliteal artery and vein and part of the popliteal nerve, surgical resection was considered risky, and we opted to simply observe her. However, owing to rapid growth of the mass and the worsening of symptoms, she underwent complete resection at 2 years and 6 months of age. A histological examination confirmed the diagnosis of a lipoblastoma. She was discharged from our hospital 3 days after surgery with no symptoms. She could walk without pain at the 6-month follow-up, and no local recurrence was observed.

**Conclusions:**

We successfully treated a giant popliteal lipoblastoma without complications by performing a total resection. Our report provides evidence that lipoblastomas should be considered for surgical resection when they progress or symptoms appear.

## Background

Lipoblastomas (LBSs) are rare benign tumors, accounting for less than 1% of childhood neoplasms [1]. They arise from embryonic white fat and almost always occur in babies and children (approximately 80% before 3 years of age and 40% before 1 year of age) [2]. Approximately 46% of LBSs are found in the trunk and 27% in the extremities [3]. Treatment of LBS requires complete resection and, owing to recurrence rates of 14.3 to 25% [1], careful follow-up for 3 to 5 years after surgery [4, 5]. Here we present a case of a 23-month-old Japanese girl with a giant popliteal LBS that was successfully treated via complete resection. At the most recent follow-up, 6 months after the operation, no local recurrence or symptoms was observed.

## Case presentation

Our patient was a 23-month-old Japanese girl. She was born full-term after a normal pregnancy and delivery. At 6 months of age, she presented at a nearby hospital with a mass on the popliteal side of her lower right leg, which was diagnosed as a benign adipose tumor; the diagnosis was made via magnetic resonance imaging (MRI) because she had no symptoms. The mass gradually increased in size, and she had difficulty in walking.

**Fig. 1:**
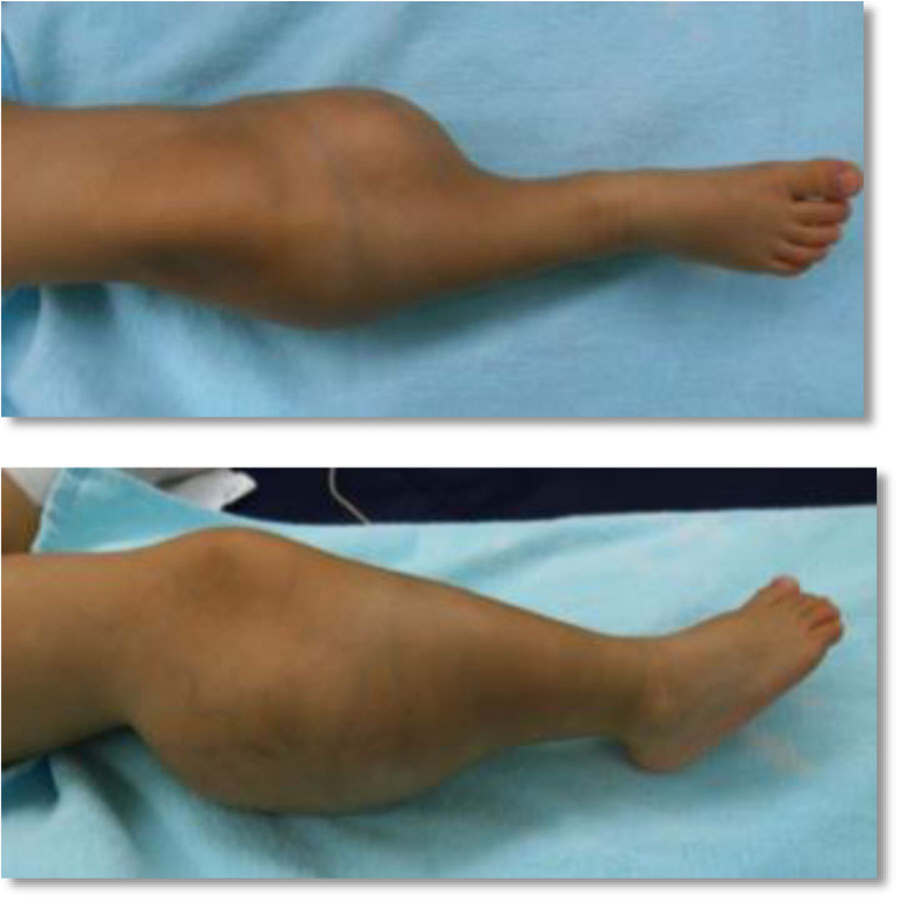
Physical findings at the first visit of the patient to our hospital. There was a subcutaneous mass on the popliteal side of lower right leg, 13 × 10 × 7 cm in size. Two different views are shown

**Fig. 2:**
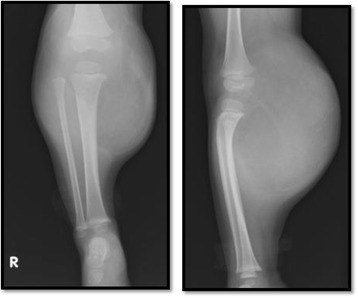
Radiography findings. Anteroposterior (*left*) and lateral (*right*) radiographs showed a localized mass on the popliteal side of lower right leg and proximal tibia deformity

**Fig. 3:**
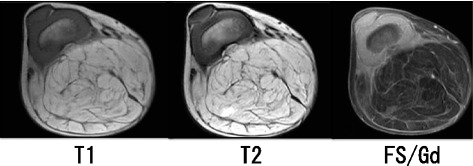
Magnetic resonance findings. The mass appeared hyperintense on T1-weighted (*left*) and T2-weighted images (*middle*) but not on fat-saturated T2-weighted images. Numerous internal fibrous partitions were visible on gadolinium-enhanced fat-saturated images (*right*). *FS* fat-saturated, *Gd* gadolinium-enhanced

At 1 year and 11 months of age, she was referred to our hospital. A physical examination revealed a subcutaneous mass on the popliteal side of her lower right leg (Fig. 1). The mass was soft, smooth, painless, immobile, and 13 × 10 × 7 cm in size. Knee movement was restricted because of the tumor, but there were no neurological symptoms. Laboratory parameters including tumor marker levels were within normal ranges. Radiography showed a localized mass on the popliteal side of her lower right leg, with compression of the mass causing proximal tibia deformity (Fig. 2). MRI showed a giant circumscribed subcutaneous mass that was hyperintense on T1-weighted and T2-weighted images but not fat-saturated on T2-weighted images (Fig. 3). The mass had numerous internal fibrous partitions that were visible on gadolinium-enhanced fat-saturated images. Based on these findings, she was diagnosed as having an LBS.

**Fig. 4:**
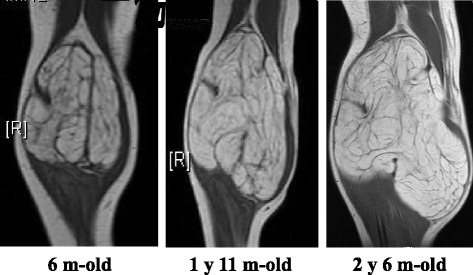
Magnetic resonance images showing the clinical course. The mass gradually increased in size from 6 months to 1 year and 6 months of age, after which it grew rapidly, eventually preventing the patient from walking. *m* month, *y* year

**Fig. 5:**
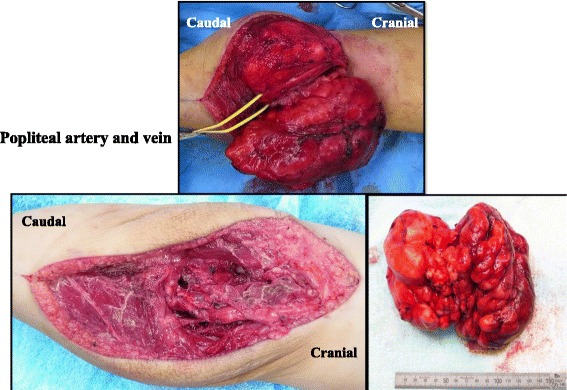
Operative findings. Because the mass surrounded the popliteal artery and vein, we divided it at these vessels, so that we could remove it without leaving any visible tumor tissue

Surgical resection was considered but deemed risky and technically challenging because the mass surrounded her popliteal artery and vein and part of the popliteal nerve, as revealed by MRI. We decided to observe only, as the mass was not expected to grow. However, it grew rapidly (Fig. 4) and her symptoms worsened within the year after our initial examination; hence, she underwent complete resection at 2 years and 6 months of age (Fig. 5). Because the mass surrounded her popliteal artery and vein, we divided it at these vessels, which allowed us to remove it without leaving any observable tumor tissue and without sacrifice of her neurovascular structures.

**Fig. 6:**
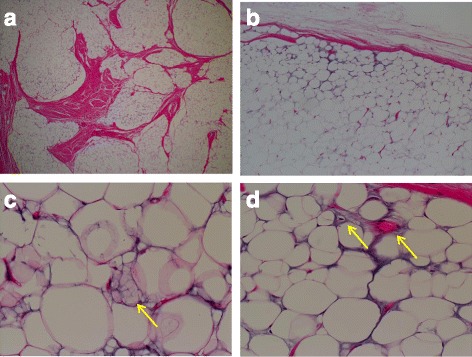
Histological findings. Internal fibrous partitions created lobular cysts (**a**). Abundant mature fatty cells in the center of tumor (**b**) surrounded by immature lipoblasts (**c**) and vascular and primitive mesenchymal cells (**d**). The *yellow arrows* indicate immature lipoblasts (**c**) and mesenchymal cells (**d**)

A histological examination of the tumor showed that it mainly consisted of mature fatty cells, fibrous partitions that created lobular cysts, immature lipoblasts, primitive mesenchymal cells, and stroma (Fig. 6). These findings confirmed the preoperative diagnosis of LBS. Our patient was discharged from our hospital 3 days after surgery without any residual symptoms. She could walk without pain at the 6-month follow-up, and no local recurrence was observed on magnetic resonance images.

## Discussion

As defined by Jaffe in 1926, LBSs are atypical benign lipomatous lesions that consist of cells resembling embryonic white fat [6]. They differ from lipomas, which are more common and do not contain lipoblasts, and almost always occur in babies and children, with a male to female ratio of 3.8:1. Typical locations include the trunk and extremities. There are two types: type 1 LBSs (80%) are subcutaneous and localized, with no invasion of nearby tissue, whereas type 2 LBSs (20%) invade deeply, progress rapidly, and have a high risk of recurrence [1, 2, 4, 5]. Neither type malignantly transforms or metastasizes.

On radiography, an LBS appears only as a shadow of soft tissue, without calcification or specific features. MRI shows a lobulated fatty mass with various amounts of non-enhancing cystic areas and internal fibrous partitions intermixed with a few small enhancing soft tissue nodules [7]. The histological findings mirror the radiological findings: the tumor contains internal fibrous partitions with abundant mature fatty cells at its center surrounded by immature lipoblasts, vascular and primitive mesenchymal cells, stroma, and other components. Mature fatty cells tend to be non-enhancing on MRI, whereas the internal fibrous partitions and other components are enhancing.

Lipomas do not contain internal fibrous partitions, which allows us to distinguish them from LBSs on MRI. Myxoid liposarcomas have internal fibrous partitions and myxoid areas as do LBSs; however, they occur in middle-aged and elderly patients, whereas LBSs mainly occur in children less than 3-years old.

Recently identified cytogenetic abnormalities may facilitate the diagnosis of LBSs. It is now clear that LBSs harbor a chromosome 8 rearrangement; the region containing the *PLAG1* oncogene on chromosome 8q12 is rearranged in 70% of LBSs, and chromosome 8 polysomy is observed in 18% of LBSs [3, 8–10]. The *PLAG1* gene is involved in the development of the central neurological system [3]. The sensitivity of *PLAG1* rearrangement for diagnosing LBS is 77%, and the specificity is 98% [9, 10]. Myxoid liposarcomas are characterized by translocations of chromosomes 12 and 16 and thus are distinct from LBSs in terms of genetic alterations [3].

The only treatment for LBS is complete surgical resection [3–5]. No benefits of chemotherapy or radiation have been reported. The recurrence rate after surgery is relatively high (14.3 to 25%) for type 2 LBSs, which deeply invade tissue and progress rapidly. Moreover, margins are positive after surgery in 42% of cases [3]. To avoid recurrence, removal of all tumor tissue is critical. When surgical margins are difficult to determine, rapid pathology during surgery should be performed. Although recurrence is usually focal and not metastatic, an additional operation should be considered [2].

When a patient with an LBS has no symptoms, the surgeon may decide to observe the patient rather than perform surgery. There are two reasons for doing so. First, surgery performed in young children is risky and requires considerable expertise. Second, LBSs are benign, and if their progression does not occur, they will likely become lipomas, with all immature lipoblasts becoming mature fatty cells. When the mass progresses rapidly during a prescribed period and functional symptoms appear, surgical treatment should be considered.

After surgery, careful routine observation by MRI is required. Because tumors can recur as long as 84 months postoperatively, a follow-up period of at least 3 to 5 years is recommended, with MRI performed at fixed intervals [4, 5].

## Conclusions

We successfully treated a giant popliteal LBS in a child without complications by performing a total resection. There was no local recurrence at the 6-month follow-up as assessed by MRI. LBSs are benign tumors that can progress rapidly and cause numerous symptoms without a spontaneous resolution. Therefore, if possible, surgical resection should be the treatment of choice for LBSs when the mass progresses or symptoms appear.

## Abbreviations

LBS: Lipoblastoma
MRI: Magnetic resonance imaging
